# Drug resistance of *Mycobacterium tuberculosis* in Malawi: a cross-sectional survey

**DOI:** 10.2471/BLT.13.126532

**Published:** 2014-09-18

**Authors:** Michael Abouyannis, Russell Dacombe, Isaias Dambe, James Mpunga, Brian Faragher, Francis Gausi, Henry Ndhlovu, Chifundo Kachiza, Pedro Suarez, Catherine Mundy, Hastings T Banda, Ishmael Nyasulu, S Bertel Squire

**Affiliations:** a Centre for Applied Health Research & Delivery, Department of Clinical Sciences, Liverpool School of Tropical Medicine, Pembroke Place, Liverpool, L3 5QA, England.; b National Tuberculosis Control Programme, Lilongwe, Malawi.; c Research for Equity and Community Health Trust, Lilongwe, Malawi.; d Tuberculosis Control Assistance Programme, Management Sciences for Health, Lilongwe, Malawi.; e Management Sciences for Health, Arlington, United States of America.; f World Health Organization, Lilongwe, Malawi.

## Abstract

**Objective:**

To document the prevalence of multidrug resistance among people newly diagnosed with – and those retreated for – tuberculosis in Malawi.

**Methods:**

We conducted a nationally representative survey of people with sputum-smear-positive tuberculosis between 2010 and 2011. For all consenting participants, we collected demographic and clinical data, two sputum samples and tested for human immunodeficiency virus (HIV).The samples underwent resistance testing at the Central Reference Laboratory in Lilongwe, Malawi. All *Mycobacterium tuberculosis* isolates found to be multidrug-resistant were retested for resistance to first-line drugs – and tested for resistance to second-line drugs – at a Supranational Tuberculosis Reference Laboratory in South Africa.

**Findings:**

Overall, *M. tuberculosis* was isolated from 1777 (83.8%) of the 2120 smear-positive tuberculosis patients. Multidrug resistance was identified in five (0.4%) of 1196 isolates from new cases and 28 (4.8%) of 581 isolates from people undergoing retreatment. Of the 31 isolates from retreatment cases who had previously failed treatment, nine (29.0%) showed multidrug resistance. Although resistance to second-line drugs was found, no cases of extensive drug-resistant tuberculosis were detected. HIV testing of people from whom *M. tuberculosis* isolates were obtained showed that 577 (48.2%) of people newly diagnosed and 386 (66.4%) of people undergoing retreatment were positive.

**Conclusion:**

The prevalence of multidrug resistance among people with smear-positive tuberculosis was low for sub-Saharan Africa – probably reflecting the strength of Malawi’s tuberculosis control programme. The relatively high prevalence of such resistance observed among those with previous treatment failure may highlight a need for a change in the national policy for retreating this subgroup of people with tuberculosis.

## Résumé

**Objectif:**

Documenter la prévalence de la résistance polymédicamenteuse de la tuberculose parmi les personnes nouvellement diagnostiquées et les personnes traitées à nouveau au Malawi.

**Méthodes:**

Nous avons mené une enquête nationale représentative des personnes atteintes de tuberculose à frottis d'expectoration positif entre 2010 et 2011. Pour tous les participants consentants, nous avons recueilli les données démographiques et cliniques, deux échantillons d'expectoration et effectué le dépistage du virus de l'immunodéficience humaine (VIH). Les échantillons ont subi des tests de résistance au Laboratoire central de référence de Lilongwe, au Malawi. Tous les isolats de *Mycobacterium tuberculosis* qui ont présenté une résistance polymédicamenteuse ont été retestés pour la résistance aux médicaments de première intention - et testés pour la résistance aux médicaments de deuxième intention – dans un laboratoire de référence supranational pour la tuberculose en Afrique du Sud.

**Résultats:**

Dans l'ensemble, *M. tuberculosisM. tuberculosis* a été isolé chez 1777 (83,8%) des 2120 patients atteints de tuberculose à frottis positif. La résistance polymédicamenteuse a été identifiée dans 5 (0,4%) des 1196 isolats obtenus à partir des nouveaux cas et dans 28 (4,8%) des 581 isolats obtenus à partir des personnes qui recevaient à nouveau un traitement. Parmi les 31 isolats issus des cas retraités qui ont connu un échec de traitement, 9 (29%) isolats ont présenté une résistance polymédicamenteuse . Bien que la résistance aux médicaments donnés en deuxième intention ait été identifiée, aucun cas de tuberculose ultrarésistante aux médicaments n'a été détecté. Les dépistages du VIH des personnes à partir desquelles les isolats de *M. tuberculosisM. tuberculosis* ont été obtenus ont montré que 577 (48,2%) des personnes nouvellement diagnostiquées et 386 (66,4%) des personnes recevant à nouveau le traitement étaient séropositives.

**Conclusion:**

La prévalence de la résistance polymédicamenteuse chez les personnes atteintes de tuberculose à frottis positif était faible en Afrique subsaharienne – reflétant probablement la force du programme de contrôle de la tuberculose du Malawi. La prévalence relativement élevée de cette résistance observée chez les personnes pour lesquelles le traitement précédent a échoué peut mettre en évidence un besoin de changement dans la politique nationale en matière de retraitement de ce sous-groupe de personnes atteintes de tuberculose.

## Resumen

**Objetivo:**

Documentar la prevalencia de la resistencia a medicamentos múltiples entre pacientes a quienes se ha diagnosticado recientemente o han vuelto a recibir tratamiento para la tuberculosis en Malawi.

**Métodos:**

Llevamos a cabo una encuesta representativa a nivel nacional de pacientes con tuberculosis que dieron positivo en el análisis de esputo entre 2010 y 2011. Para todos los participantes adultos, se recogieron datos demográficos y clínicos, dos muestras de esputo y realizamos pruebas del virus de inmunodeficiencia humana (VIH). Las muestras se sometieron a pruebas de resistencia en el Laboratorio Central de Referencia en Lilongwe (Malawi). Se volvieron a examinar todas las cepas de *Mycobacterium tuberculosis* multirresistentes para probar la resistencia a los medicamentos de primera línea y se probó su resistencia a los medicamentos de segunda línea en un laboratorio de referencia supranacional para la tuberculosis en Sudáfrica.

**Resultados:**

En general, la *M. tuberculosisM. tuberculosis* se aisló en 1777 (83,8%) de los 2120 pacientes de tuberculosis con baciloscopia positiva. Se detectó multirresistencia a medicamentos en cinco (0,4%) de las 1196 cepas de casos nuevos y en 28 (4,8%) de las 581 cepas de pacientes que se volvieron a someter al tratamiento. De las 31 cepas de casos de repetición del tratamiento que no habían respondido previamente al tratamiento, nueve (29,0%) mostraron multirresistencia a medicamentos. Pese a que se halló resistencia a los medicamentos de segunda línea, no se detectaron casos de tuberculosis con resistencia extendida a medicamentos. Las pruebas del VIH de quienes se obtuvieron cepas de *M. tuberculosisM. tuberculosis* mostraron que 577 (48,2%) de los pacientes con diagnóstico reciente y 386 (66,4%) de los pacientes que se volvieron a someter al tratamiento dieron positivo.

**Conclusión:**

La prevalencia de la multirresistencia a medicamentos entre los pacientes con tuberculosis que dieron positivo en la baciloscopia positiva fue baja en el África subsahariana, lo cual probablemente refleja la eficacia del programa de control de la tuberculosis de Malawi. La prevalencia relativamente alta de dicha resistencia observada entre los pacientes que no respondieron al tratamiento anterior puede poner de manifiesto la necesidad de un cambio en la política nacional para volver a tratar a este subgrupo de pacientes con tuberculosis.

## ملخص

**الغرض:**

توثيق انتشار مقاومة الأدوية المتعددة بين الأشخاص الذين جرى تشخيص إصابتهم حديثاً بالسل – والذين تكرر علاجهم من السل - في ملاوي.

**الطريقة:**

أجرينا دراسة استقصائية تمثيلية على الصعيد الوطني للأشخاص المصابين بالسل وسجلوا نتائج إيجابية لاختبار لطاخة البلغم بين 2010 و2011. وقمنا بجمع البيانات الديمغرافية والسريرية وعينتين من البلغم واختبارهما لتحديد الإصابة بفيروس العوز المناعي البشري من جميع المشاركين الذين أبدوا موافقتهم. وتم إجراء اختبار المقاومة للعينات في المختبر المرجعي المركزي في ليلونغوي، بملاوي. وتم تكرار اختبار جميع مستفردات البكتريا المتفطرة السليّة التي تبين مقاومتها للأدوية المتعددة من أجل تحديد مقاومتها لأدوية الخط الأول – واختبارها من أجل تحديد مقاومتها لأدوية الخط الثاني – في مختبر مرجعي للسل على الصعيد فوق الوطني في جنوب أفريقيا.

**النتائج:**

بشكل عام، تم استفراد البكتريا المتفطرة السليّة من 1777 (83.8 %) مريضاً من أصل 2120 مريضاً بالسل سجلوا نتائج إيجابية لاختبار اللطاخة. وتم تحديد مقاومة الأدوية المتعددة في خمس (0.4 %) من أصل 1196 مستفردة من حالات جديدة و28 (4.8 %) من أصل 581 مستفردة من الأشخاص الذين تكرر علاجهم. وأظهرت تسع (29.0 %) من أصل 31 مستفردة من الحالات التي تكرر علاجها بعد فشل علاجها في السابق مقاومة للأدوية المتعددة. وعلى الرغم مما تبين من مقاومة لأدوية الخط الثاني، لم يتم اكتشاف حالات للسل الشديد المقاوم للأدوية. وتبين من اختبار فيروس العوز المناعي البشري للأشخاص الذين تم الحصول على مستفردات البكتريا المتفطرة السليّة منهم أن 577 (48.2 %) من الأشخاص الذين جرى تشخيصهم حديثاً و386 (66.4 %) من الأشخاص الذين تكرر علاجهم سجلوا نتائج إيجابية.

**الاستنتاج:**

كان انتشار مقاومة الأدوية المتعددة بين الأشخاص المصابين بالسل الذين سجلوا نتائج إيجابية لاختبار اللطاخة منخفضاً في أفريقيا جنوب الصحراء الكبرى – بما يوضح على نحو محتمل قوة برنامج مكافحة السل في ملاوي. من المحتمل أن يؤكد الارتفاع النسبي لانتشار هذه المقاومة التي لوحظت بين الأشخاص الذين فشل علاجهم في السابق على الحاجة للتغيير في السياسة الوطنية من أجل تكرار علاج هذه الفئة الفرعية من الأشخاص المصابين بالسل.

## 摘要

**目的:**

记录马拉维肺结核新诊以及复治人群多耐药性流行率。

**方法:**

我们针对2010年和2011年之间痰涂片阳性肺结核患者进行了具有全国代表性的调查。对于所有的参与者，我们都收集了人口和临床数据、两份唾液样本并进行艾滋病毒（HIV）检测。这些样本在马拉维隆圭中央参考实验室接受耐药性检测。在南非超国家结核病参考实验室对发现具有多耐药性的所有分枝杆菌肺结核分离菌再次进行一线药物的耐药性检测，然后进行二线药物耐药性检测。

**结果:**

总的来说，在2120名痰涂片阳性肺结核患者中，从1777名（83.8%）患者中分离出肺结核分枝杆菌。从新患者的1196个分离菌中确定了5个（0.4%）有多耐药性，从复治患者的581个分离菌中确定了28个（4.8%）有多耐药性。在曾经治疗失败的复治病例中获得的31个分离菌中，9个（29.0%）显示出多耐药性。尽管发现二线药物耐药性，但未发现广泛耐药性的肺结核病例。对获得肺结核分枝杆菌分离菌人群的艾滋病毒检测显示，新患者中有577例（48.2%）为阳性，复治患者中有386例（66.4%）为阳性。

**结论:**

撒哈拉以南非洲痰涂片阳性肺结核患者多耐药性的流行率较低——可能反映了马拉维的肺结核病控制规划的效力。在先前治疗失败的人群中观察这种耐药性的流行率相对较高，这可能凸显了对这个肺结核患者子群的全国性复治政策作出改变的需求。

## Резюме

**Цель:**

Задокументировать распространенность множественной лекарственной устойчивости при первичном и повторном лечении больных туберкулезом в Малави.

**Методы:**

В 2010-2011 гг. было проведено национальное репрезентативное исследование больных туберкулезом легких с бактериовыделением. У всех согласившихся принять участие в исследовании были собраны демографические и клинические данные и взяты два образца мокроты; кроме того, они прошли тестирование на вирус иммунодефицита человека (ВИЧ). Лекарственная устойчивость полученных образцов была проверена в Центральной референс-лаборатории г. Лилонгве, Малави. Все изоляты микобактерий туберкулеза с выявленной множественной лекарственной устойчивостью были подвергнуты дополнительному тестированию на устойчивость к лекарственным препаратам первой и второй линии в наднациональной туберкулезной референс-лаборатории в Южной Африке.

**Результаты:**

В итоге, наличие *микобактерий туберкулеза* было выявлено у 1777 (83,8%) из 2120 больных туберкулезом легких с бактериовыделением. Множественная лекарственная устойчивость была обнаружена у пяти (0,4%) из 1196 изолятов, взятых у лиц, получавших первичное лечение туберкулеза, и у 28 (4,8%) из 581 изолятов, взятых у лиц, получавших повторное лечение. Из изолятов, взятых у лиц, получавших повторное лечение после неудачного первичного, множественная лекарственная устойчивость была выявлена в девяти (29%) случаях из 31. Притом, что устойчивость к лекарствам второй линии была обнаружена, случаев туберкулеза с широкой лекарственной устойчивостью выявлено не было. Тестирование на ВИЧ лиц, у которых были выделены изоляты микобактерий туберкулеза, показало наличие вируса у 577 (48,2%) больных, у которых туберкулез был выявлен впервые, и у 386 (66,4%) больных, получавших повторное лечение.

**Вывод:**

Распространенность лекарственной устойчивости среди больных туберкулезом легких с бактериовыделением в странах Африки южнее Сахары невелика, что, по-видимому, свидетельствует об эффективности противотуберкулезной программы Малави. В то же время, довольно высокая распространенность таких случаев среди лиц, лечение которых в прошлом не принесло результата, может указывать на необходимость изменения национального подхода к повторному лечению этой подкатегории больных туберкулезом.

## Introduction

Although the World Health Organization (WHO) has monitored the emergence of drug resistance of *Mycobacterium tuberculosis* since 1994,^1^ there have been few national surveys of such resistance in sub-Saharan Africa.^2^

In 2012, it was estimated that about 1.9% of people newly diagnosed and 9.4% of those undergoing retreatment in Africa had multidrug-resistant (MDR) tuberculosis.^3^ The prevalence of MDR tuberculosis in Africa varies between countries^4^ and might be generally increasing.^3^^,^^5^

Over several years, attempts have been made – at the Central Reference Laboratory in Lilongwe – to isolate *M. tuberculosis* from all smear-positive patients undergoing retreatment in Malawi to investigate drug susceptibility. In 2008, about 8% of people investigated in this manner were found to have MDR tuberculosis (James Mpunga, Malawi National Tuberculosis Control Programme, personal communication, 2008) – although most of the samples came from urban centres and the laboratory’s attempts to isolate *M. tuberculosisM. tuberculosis* often failed.^6^ The only published data on MDR tuberculosis in Malawi indicated that just 0.5% of people newly diagnosed with tuberculosis and 0.9% of people being retreated in Karonga district had MDR tuberculosis in 1996–1998.^7^

In 2007, the nationally recommended treatment regimen for people newly diagnosed with tuberculosis in Malawi changed. The initial supervised treatment remained the same – i.e. daily isoniazid, rifampicin, pyrazinamide and ethambutol for 2 months – but the unsupervised continuation phase changed from 6 months of isoniazid and ethambutol to 4 months of isoniazid and rifampicin.^8^^,^^9^ There are four problems since this change that need monitoring. The first is that poor adherence during this currently-recommended continuation phase could lead to the emergence of MDR tuberculosis. Another problem is that nothing is known about the resistance of Malawian isolates of *M. tuberculosis* to the second-line drugs that began to be used routinely in Malawi in 2007. A third problem is the high prevalence of human immunodeficiency virus (HIV) infection among people with tuberculosis.^10^ In 2010, 63% of Malawian tuberculosis patients tested for HIV were found positive.^4^ Finally, the national prevalence of drug-resistant tuberculosis may be affected by migration of people from neighbouring countries, where such outbreaks have occurred.^11^ Given these issues, we conducted a national survey of resistance to anti-tuberculosis drugs in Malawi.

## Methods

### Study setting and design

We engaged all of Malawi’s 48 tuberculosis registration centres to conduct a prospective, cross-sectional survey. The centres were grouped into three zones – northern, central and southern – for phased sample collection.

### Data collection and management

Health workers in each registration centre formed a recruitment team and attended a three-day training course about the survey protocol. They subsequently collected data on each consenting smear-positive tuberculosis patient, including the patient’s age, sex, level of education, occupation, marital status and HIV status – if known – and details of any previous tuberculosis treatment. After each patient was asked if they had received tuberculosis treatment, the patient’s medical records at the health facility of recruitment were checked for evidence of such treatment.

Following national policy in Malawi,^8^ each participant in the survey was offered HIV testing and counselling. At the time of the survey, two rapid blood tests – Uni-Gold Recombigen HIV-1/2 (Trinity Biotech, Bray, Ireland) and Determine HIV-1/2 (Alere, Waltham, United States of America) were used in the registration centres. Any samples giving inconclusive results were sent to the Central Reference Laboratory for retesting.

Data were collected on piloted forms and double-entered into an Epi Info (Centers for Disease Control and Prevention, Atlanta, United States of America) spreadsheet.

### Participants and case definitions

Using the definitions recommended by WHO,^12^ new cases were defined as people who had never been treated for tuberculosis – or had previously received anti-tuberculosis medications for less than one month – and retreatment cases were defined as those who had previously received tuberculosis treatment for at least one month. Retreatment cases were grouped according to the outcome of previous treatment: cured, completed, defaulted or failed. A patient was defined as cured when the person was smear-negative at, or one month before, treatment completion and on at least one previous occasion. A completed treatment was defined as a patient who completed treatment but without smear microscopy proof of cure. Persons who had treatment interruption for two consecutive months or more were grouped as defaulted. Those who remained smear-positive when tested five or six months after initiation of their previous treatment were defined as treatment failures.

For our survey, sputum samples were collected from each newly-diagnosed person with sputum-smear-positive tuberculosis seen at a registration centre in the northern, central and southern zones in May–July 2010, August–October 2010 and November 2010–January 2011, respectively. Sputum samples were also collected from each person with smear-positive tuberculosis undergoing retreatment at any registration centre between February 2010 and March 2011.

### Drug resistance definition

Isolates of *M. tuberculosis* were defined as MDR if they were at least resistant to isoniazid and rifampicin, and extremely drug resistant (XDR) if they were also resistant to an injectable drug and a quinolone of the second-line medications.

### Sample size projections

Assuming that 1.8% and 20% of the people newly diagnosed would have MDR tuberculosis and be lost to follow-up, respectively, we estimated that we needed to enrol 1260 new cases to estimate the prevalence of MDR tuberculosis among such cases with a precision of ± 1%. Similarly, assuming that 5.0% and 20% of our retreatment sample would have MDR tuberculosis and be lost to follow-up, respectively, we estimated that we would have to enrol 770 people undergoing retreatment to estimate the prevalence of MDR tuberculosis with a precision of ± 2.0%.

### Laboratory procedures

Prior to enrolment, each participant had been found positive for tuberculosis by the microscopic examination of three smears of sputum.^12^^,^^13^ Each month, a random selection of sputum smears from the registration centres – five from each health centre and 25 from each district hospital – was re-examined by a visiting laboratory supervisor. Concordance between the registration centres’ results and the supervisor’s remained above 96% during our survey.

For our survey, two additional sputum samples were collected – under supervision and approximately one hour apart – from each enrolled patient and stored at 2–8 °C in the registration centre. Efforts were made to ensure that these samples were collected before anti-tuberculosis treatment was commenced. The samples were transported to the Central Reference Laboratory, in cooler boxes, by bus or in a district health vehicle or study team vehicle.

Once a sample had reached the laboratory, it was decontaminated and further homogenized.^14^ Part of the pellet produced by centrifuging the sample was smeared, stained with auramine phenol stain and then checked for acid-fast bacilli. Another part was inoculated into two tubes of Lowenstein–Jensen medium – one containing glycerol and the other containing sodium pyruvate – which were examined for growth weekly for up to 8 weeks. Each contaminated culture was discarded and replaced with a new culture that was set up using another part of the relevant pellet – which had been kept in a refrigerator. The Capilia tuberculosis test^15^ was used to identify isolates belonging to the *M. tuberculosis* complex. Indirect susceptibility testing to isoniazid, rifampicin, ethambutol and streptomycin was performed, on one isolate per participant, using the proportion method on Lowenstein–Jenson medium.^16^

All isolates defined as MDR tuberculosis were sent to the South African Medical Research Council’s Supranational Reference Laboratory in Pretoria. There, they were retested for their susceptibility to first-line drugs – using a line probe assay and automated liquid culture^17^^,^^18^ – and tested for their susceptibility to the second-line drugs amikacin, kanamycin, capreomycin, ofloxacin and ethionamide – using automated liquid culture.

### Statistical analysis

For our final analysis, we excluded those cases from which *M. tuberculosis* was not isolated in culture. Categorical and non-parametric continuous variables were compared using χ^2^ and Wilcoxon rank-sum tests, respectively. Data on new tuberculosis cases were analysed independently from retreatment cases. Associations between MDR tuberculosis and patient age, sex, HIV status, year of previous tuberculosis treatment and outcome of previous tuberculosis treatment were compared using Poisson logistic regression analysis. Unadjusted and adjusted incidence rate ratios (IRRs) were calculated in univariate and multivariate analyses, respectively. Stata 10.0 (StataCorp. LP, College Station, United States of America) was used for the statistical analysis.

### Ethical considerations

Ethical approval was granted by the Malawi National Health Sciences Research Committee in April 2009. This study commenced in 2009, before requirements for review of all WHO-supported research by the WHO research ethics review committee were fully implemented. Written informed consent was obtained from adult participants and the caregivers of child participants. As recommended by the relevant national guidelines,^8^ all cases of MDR tuberculosis were given six months of capreomycin, levofloxacin, ethionamide, cycloserine and pyrazinamide followed by 18 months of levofloxacin, ethionamide and cycloserine.

## Results

**Fig. 1:**
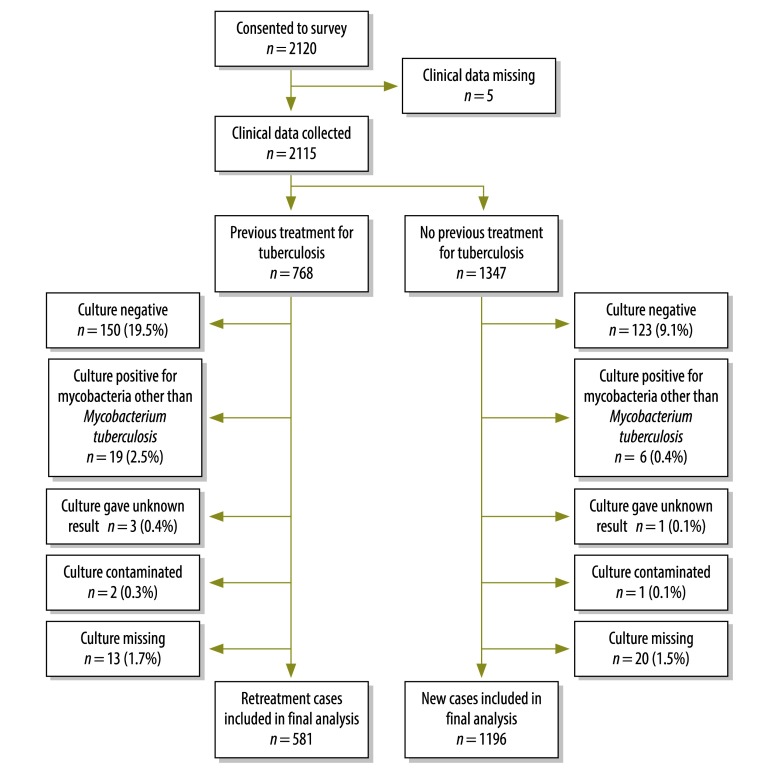
Flowchart to determine multidrug resistance in people diagnosed with tuberculosis in Malawi, 2010–2011

During the study period, 2120 smear-positive individuals consented to participate. Five were excluded as their baseline data were missing, another 1347 were classified as newly diagnosed with tuberculosis and the remaining 768 were classified as retreatment cases (Fig. 1). *M. tuberculosis* was isolated from 1196 (88.8%) of the new cases. There was no difference in the distribution of age, sex, region or HIV status between these and new cases from which *M. tuberculosis* was not isolated. *M. tuberculosis* was isolated from 581 (75.7%) of people undergoing retreatment. Those in whom *M. tuberculosis* was not isolated were older than the other retreatment cases, with mean ages of 40.7 and 36.4 years, respectively.

Compared with the new cases, people undergoing retreatment were more frequently found to be culture-negative or to be culture-positive for mycobacteria other than *M. tuberculosis*.

Of 86 treatment failures, 31 samples were culture-positive for *M. tuberculosis*, six were culture-positive for other mycobacteria and 49 were culture-negative.

The median transit time of all samples, from collection to arrival at the Central Reference Laboratory was 4 days (interquartile range, IQR: 2–7 days). Transit time had no apparent effect on the probability that a sample would be found culture-positive for *M. tuberculosis* (*P* = 0.71).

### Culture-positive tuberculosis

Culture-positive individuals in both new and retreatment groups were similar in terms of their sociodemographic characteristics (Table 1).

**Table 1:** Characteristics of people newly-diagnosed with, and retreated for, tuberculosis, Malawi, 2010–2011

Characteristic	New cases (*n* = 1196)			Retreatment cases (*n* = 581)	
	No.	% (95% CI)		No.	% (95% CI)
**Mean age (years)**	1196	35.6 (34.8–36.4)		581	36.4 (35.5–37.4)
**Sex (% male)**	1196	53.7 (50.8–56.5)		581	60.6 (56.6–64.6)
**Marital status (%)**					
Married	750	63.2 (60.5–66.0)		346	60.3 (56.3–64.3)
Single	232	19.6 (17.3–21.8)		106	18.5 (15.3–21.7)
Divorced	103	8.7 (7.1–10.3)		70	12.2 (9.5–14.9)
Widowed	101	8.5 (6.9–10.1)		52	9.1 (6.7–11.4)
**Occupation (%)**					
Business	226	19.5 (17.2–21.8)		120	21.1 (17.8–24.5)
Formal employment	195	16.8 (14.7–19.0)		126	22.2 (18.8–25.6)
Subsistence farmer	330	28.4 (25.8–31.0)		148	26.1 (22.4–29.7)
Unemployed	409	35.3 (32.5–38.0)		174	30.6 (26.8–34.4)
**Educational level achieved (%)**					
Tertiary	23	2.0 (1.2–2.8)		13	2.3 (1.0–3.5)
Secondary	262	22.5 (20.1–24.9)		171	29.9 (26.1–33.7)
Primary	729	62.6 (59.8–65.4)		319	55.8 (51.7–59.9)
None	151	13.0 (11.0–14.9)		69	12.1 (9.4–14.7)
**HIV status (%)**					
Positive	577	48.2 (45.4–51.1)		386	66.4 (62.6–70.3)
Negative	474	39.6 (36.9–42.4)		165	28.4 (24.7–32.1)
Unknown	145	12.1 (9.4–14.8)		30	5.2 (2.6–7.7)
**Region of residence (%)**					
Northern	115	9.6 (7.9–11.3)		85	14.6 (11.8–17.5)
Central west	283	23.7 (21.3–26.1)		108	18.6 (15.4–21.8)
Central east	135	11.3 (9.5–13.1)		46	7.9 (5.7–10.1)
South-west	359	30.0 (27.4–32.6)		207	35.6 (31.7–39.5)
South-east	304	25.4 (22.9–27.9)		135	23.2 (19.8–26.7)
**Outcome of previous treatment (%)**					
Cured^a^	NA	NA		389	67.0 (63.1–70.8)
Completed^b^	NA	NA		104	17.9 (14.8–21.0)
Defaulted^c^	NA	NA		49	8.4 (6.2–10.7)
Failed^d^	NA	NA		31	5.3 (3.5–7.2)
Unknown	NA	NA		8	1.4 (0.4–2.3)
**Smear score (%)^e^**					
Scanty	101	8.6 (7.0–10.2)		66	11.6 (8.9–14.2)
1+	135	11.5 (9.6–13.3)		66	11.6 (8.9–14.2)
2+	295	25.0 (22.6–27.5)		115	20.1 (16.8–23.4)
3+	647	54.9 (52.1–57.8)		324	56.7 (52.7–60.8)

CI: confidence interval; HIV: human immunodeficiency virus; NA: not applicable.

^a^ Cured defined as a smear-positive patient who was smear-negative at, or one month before, treatment completion and on at least one previous occasion.^12^

^b^ Treatment completed defined as a patient who completed treatment but without smear microscopy proof of cure.^12^

^c^ Defaulted defined as treatment interruption for two consecutive months or more.^12^

^d^ Failed defined as remaining smear-positive when tested five or six months after initiation of previous treatment.^12^

^e^ Smear scores indicate the density of acid-fast bacilli seen on a sputum smear.^13^

Note: Data are missing for some characteristics. The sum of the percentages for some characteristics may not equal 100 due to rounding.

Overall, 66.4% (386) of the retreatment cases and 48.2% (577) of the new cases were known or found to be infected with HIV, demonstrating a significantly higher HIV prevalence among people retreated (*P* < 0.01). The retreatment cases reported that they had received tuberculosis treatment between 1978 and 2010 with a median of 2.4 years (IQR: 1.1–5.9 years) before their enrolment. Just 31 (5.3%) of the culture-positive retreatment cases had failed their previous treatment (Table 1).

Among the 1196 *M. tuberculosis* isolates from new cases, ethambutol, isoniazid, rifampicin and streptomycin resistance was present in 0.5%, 3.2%, 0.8% and 4.2%, respectively (Table 2). The corresponding values for the 581 isolates from the retreatment cases were all higher (Table 2).

**Table 2:** Resistance to first-line anti-tuberculosis drugs among *Mycobacterium tuberculosis* isolates, Malawi, 2010–2011

Resistance	Isolates from new cases (*n* = 1196)			Isolates from retreatment cases (*n* = 581)	
	No.	% (95% CI)		No.	% (95% CI)
**Fully sensitive**	1116	93.3 (91.7–94.7)		470	80.9 (77.5–84.0)
**Any resistance^a^**					
R	9	0.8 (0.4–1.4)		38	6.5 (4.7–8.9)
H	38	3.2 (2.3–4.3)		66	11.4 (8.9–14.2)
E	6	0.5 (0.2–1.1)		18	3.1 (1.9–4.9)
S	50	4.2 (3.1–5.5)		49	8.4 (6.3–11.0)
**Multidrug resistance**	5	0.4 (0.1–1.0)		28	4.8 (3.2–6.9)
RH	2	0.2 (0.0–0.6)		13	2.2 (1.2–3.8)
RHE	0	0.0 (0.0–0.3)		1	0.2 (0.0–1.0)
RHS	1	0.1 (0.0–0.5)		6	1.0 (0.4–2.2)
RHES	2	0.2 (0.0–0.6)		8	1.4 (0.6–2.7)
**Other forms of resistance**	75	6.3 (5.0–7.8)		83	14.3 (11.5–17.4)
R only	3	0.3 (0.1–0.7)		9	1.5 (0.7–2.9)
H only	22	1.8 (1.2–2.8)		32	5.5 (3.8–7.7)
E only	2	0.2 (0.0–0.6)		4	0.7 (0.2–1.8)
S only	35	2.9 (2.1–4.1)		30	5.2 (3.5–7.3)
RS	1	0.1 (0.0–0.5)		0	0.0 (0.0–0.6)
RE	0	0.0 (0.0–0.3)		1	0.2 (0.0–1.0)
HE	1	0.1 (0.0–0.5)		2	0.3 (0.0–1.2)
HS	10	0.8 (0.4–1.5)		3	0.5 (0.1–1.5)
ES	1	0.1 (0.0–0.5)		1	0.2 (0.0–1.0)
HES	0	0.0 (0.0–0.3)		1	0.2 (0.0–1.0)

CI: confidence interval; E: ethambutol; H: isoniazid; R: rifampicin; S: streptomycin.

^a^ Any resistance indicates resistance to the anti-tuberculosis medication tested, independent of resistance results to the other medications.

**Fig. 2:**
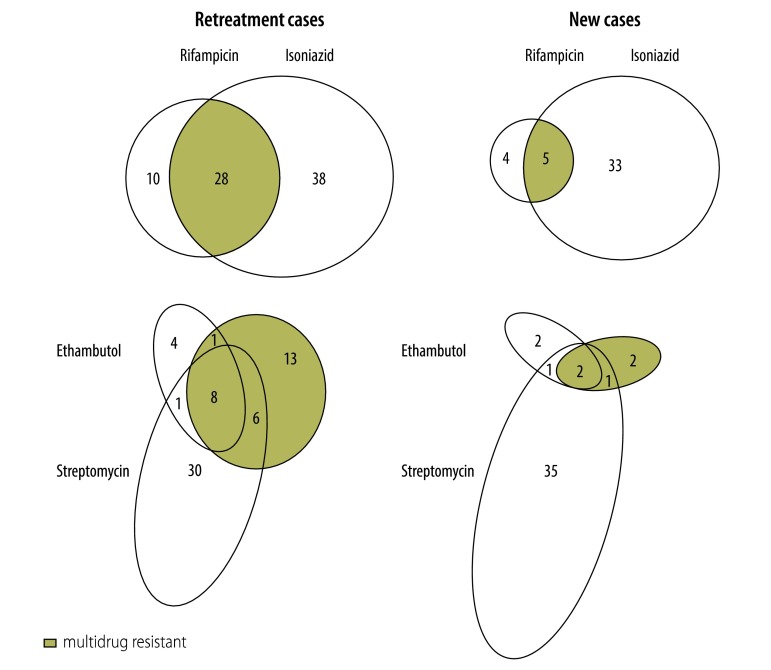
Resistance patterns of *Mycobacterium tuberculosis* to anti-tuberculosis drugs, Malawi, 2010–2011

Five (0.4%) of the 1196 new cases had MDR tuberculosis (Table 2 and Fig. 2). Other types of resistance (mono-resistance or any combination of drug resistance excluding MDR tuberculosis) were identified in 75 (6.3%) of the new cases but the remaining 1116 (93.3%) *M. tuberculosis* isolates from new cases were found to be sensitive to all four first-line drugs.

Twenty-eight (4.8%) of the 581 *M. tuberculosis* isolates from retreatment cases showed multidrug resistance (Table 2). Other types of resistance were identified in 83 (14.3%) of the retreatment cases but the remaining 470 (80.9%) *M. tuberculosis* isolates from retreatment cases were found to be sensitive to all four first-line drugs (Table 2 and Fig. 2).

In the multivariate analysis, sex, age and HIV status were not found to be significantly associated with MDR tuberculosis among new or retreatment cases. There was also no evidence of a significant association between region of residence and MDR tuberculosis. All of the 28 retreatment cases with MDR tuberculosis had received treatment in the previous five years – 23 (82%) within the previous two years. MDR tuberculosis in people undergoing retreatment was found to be significantly and inversely associated with time since previous treatment (adjusted IRR: 0.7, 95% confidence interval, CI: 0.5–0.9). Previous treatment failure – but no other previous treatment outcome – was strongly associated with MDR tuberculosis (adjusted IRR: 3.7, 95% CI: 1.6–8.4). Of the 31 treatment failures, nine (29.0%) cultured multi-drug resistant *M. tuberculosis*.

Of the 33 isolates of *M. tuberculosis* found to be multidrug-resistant in Malawi, 30 successfully underwent retesting in South Africa, and 11 of these were sensitive to either isoniazid or rifampicin or both of these drugs. If the results from South Africa are used as the gold standard, this indicates a 36.7% false-positive rate (11/30 in Table 3). When a random sample of 106 isolates of *M. tuberculosis* found not to be multidrug-resistant in Malawi were retested in South Africa, one was identified as MDR tuberculosis – giving a 0.9% false-negative rate (1/106 in Table 3).

**Table 3:** Comparison of anti-tuberculosis drug susceptibility testing of Malawian *Mycobacterium tuberculosis* isolates, 2010–2011

Malawian result	South African result^a^			
	Sensitive to all drugs	Resistant to rifampicin only	Resistant to isoniazid only	MDR
Sensitive to all drugs	87	1	2	0
Resistant to rifampicin only	2	0	0	0
Resistant to isoniazid only	6	0	7	1
MDR	4	2	5	19

MDR: multidrug-resistant.

^a^ The table shows the numbers of *M. tuberculosis* isolates, from Malawian cases of smear-positive pulmonary tuberculosis, that were tested for resistance to isoniazid, rifampicin, ethambutol and streptomycin in both the Central Reference Laboratory (Lilongwe, Malawi) and the South African Medical Research Council’s Supranational Reference Laboratory (Pretoria, South Africa).

The 20 isolates found to show multidrug resistance in South Africa were re-cultured in South Africa and tested for resistance to several second-line drugs. Although 18 of these isolates were successfully re-cultured and tested, none showed extensive drug resistance (Table 4).

**Table 4:** Resistance to second-line anti-tuberculosis drugs among 18 multidrug-resistant *Mycobacterium tuberculosis* isolates, Malawi, 2010–2011

Isolate	Resistance^a^				
	Amikacin	Kanamycin	Capreomycin	Ofloxacin	Ethionamide
1–14	susceptible	susceptible	susceptible	susceptible	resistant
15	susceptible	resistant	susceptible	susceptible	resistant
16	susceptible	susceptible	resistant	susceptible	resistant
17	resistant	resistant	resistant	susceptible	resistant
18	resistant	susceptible	resistant	susceptible	resistant

^a^ Amikacin, kanamycin, capreomycin, ofloxacin and ethionamide were tested at concentrations up to 1.0, 5.0, 2.5, 2.0 and 5.0 µg/mL, respectively.

## Discussion

This is the first national survey of anti-tuberculosis drug resistance done in Malawi. We found the prevalence of MDR tuberculosis among people newly diagnosed to be low, at 0.4%. As about 7200 new cases of smear-positive tuberculosis have occurred annually in Malawi over recent years,^4^ we can expect there to be 29 cases of primary MDR tuberculosis in Malawi annually. Although we found the prevalence of MDR tuberculosis among retreatment cases to be significantly higher, as generally observed,^19^ this could be expected to produce only 27 secondary cases of MDR tuberculosis annually.

The rates described here represent the lowest values reported in sub-Saharan Africa up to 2011.^3^ Neighbouring Mozambique identified multidrug resistance in 3.5% of new tuberculosis cases and 11.2% of retreatment cases in 2007. In 2009, Swaziland reported corresponding values of 7.7% and 33.9%, respectively.^5^ During our survey, the Central Reference Laboratory successfully isolated *M. tuberculosis* from the sputum samples from 88.8% of new cases and 75.7% of retreatment cases. Although the sample transit times recorded during our survey were disappointing, long transit times were not associated with isolation failures. Mycobacteria could not be grown from 49 of 86 samples from treatment failures, probably because the bacilli in the 49 samples were dead. Mycobacteria other than *M. tuberculosis* were cultured from six treatment failures. The proportion of sputum samples from retreatment cases that were found culture-positive for *M. tuberculosis* was significantly lower than the corresponding value for the new cases. This difference is partly explained by (i) the low isolation rate from treatment failures; (ii) the fact that samples from retreatment cases were relatively more likely to grow mycobacteria other than *M. tuberculosis*; and (iii) the fact that sputum samples from retreatment cases are relatively more likely to be collected from patients who have already begun treatment for their current episode of tuberculosis.

Since the results recorded by Malawi’s Central Reference Laboratory were associated with a 36.7% false-positivity rate and a 0.9% false-negativity rate, the prevalences of MDR tuberculosis that we recorded in Malawi – although low – could overestimate the true values. Given the laboratory’s limited capacity and the observation that resistance patterns probably do not vary between smear-positive and smear-negative cases of tuberculosis,^20^ we did not investigate the drug resistance of any *M. tuberculosis* isolates from smear-negative tuberculosis patients.

We found HIV prevalence among new smear-positive cases of pulmonary tuberculosis to be 48.2%. The HIV prevalences reported among all tuberculosis cases by Malawi’s National Tuberculosis Programme in 2010 and 2011 were higher, at 63% and 60%, respectively.^21^ The programme’s observations indicate that HIV prevalence among smear-negative cases of pulmonary tuberculosis exceeded 65% in 2010–2011. By focusing on smear-positive cases, we probably limited the extent to which we could explore associations between HIV and MDR tuberculosis. Although we found no association between HIV and MDR tuberculosis, it is possible that such an association exists in the overall population of people with tuberculosis. The existence of such a link remains a matter of controversy^3^^,^^5^^,^^22^^,^^23^ but concomitant HIV infection certainly poses some unique challenges in the management of tuberculosis.^10^

Although we collected samples from different areas of Malawi at different times of the year, a retrospective analysis of new tuberculosis case notifications between 1999 and 2007 suggested that there was little variation in the number of new cases occurring in each quarter of the year (James Mpunga, Malawi National Tuberculosis Control Programme, personal communication, 2010).

The low prevalence of MDR tuberculosis that we recorded may be attributable to the success of Malawi’s tuberculosis control programme. The frequencies of success in the treatment of tuberculosis in Malawi – 88% for new cases and 85% for retreatment cases – are among the highest recorded in sub-Saharan Africa.^4^ We recorded higher prevalences of streptomycin resistance than of rifampicin or isoniazid resistance, perhaps because streptomycin was included in the recommended first-line treatment for tuberculosis in Malawi until 1992.

We detected no XDR tuberculosis but did observe some resistance to second-line drugs. Since all of our isolates tested for resistance to ethionamide were found positive, the currently recommended 24-month regimen for the treatment of MDR tuberculosis in Malawi needs to be revised. Resistance to the second-line injectables was detected but not resistance to ofloxacin. At the time of the survey, Malawi’s Central Reference Laboratory relied entirely upon the South African Supranational Reference Laboratory for the identification of Malawian cases of XDR tuberculosis.^8^

Treatment failure – frequently a forewarning for the development of drug-resistant tuberculosis^24^ – was associated with a 29.0% risk of MDR tuberculosis in our survey. The initiation of a standard retreatment regimen while awaiting the results of drug susceptibility testing may amplify resistance in cases with pre-existing MDR tuberculosis.^25^^,^^26^ Although use of an empirical MDR treatment regimen has been suggested as a replacement for the standard retreatment regimen for all treatment failures,^24^^,^^27^^,^^28^ such a change in Malawi would expose most treatment failures – i.e. those who do not have MDR tuberculosis – to a more toxic and less effective therapy. During our survey, all patients with MDR tuberculosis who were diagnosed by phenotypic testing at the Central Reference Laboratory – including the 11 cases classified as drug sensitive when their sputum samples were investigated in South Africa – were managed with the nationally recommended second-line regimen. This was because (i) the phenotypic results were seen as more predictive of clinical response; (ii) the South African results became available several months after the patients had started second-line therapy; and (iii) it was felt that any changes to treatment made after the South African results became available would be confusing to patients.

## Conclusion

The prevalence of MDR tuberculosis is currently low in Malawi – probably as the result of a strong tuberculosis control programme – whereas HIV-coinfection, which has been associated with high mortality in the presence of drug-resistant tuberculosis, is common. Almost a third of the treatment failures we investigated had MDR tuberculosis. Given the discovery of ethionamide resistance in all 18 of the MDR tuberculosis isolates investigated for such resistance, ethionamide should be replaced with an alternative drug in Malawi’s current MDR tuberculosis treatment regimen. Given an increasing prevalence of drug resistance in some neighbouring countries and the recent introduction of unsupervised rifampicin into tuberculosis treatment regimens in Malawi, we recommend repeating this survey within three years.
